# Deletion of the Vaccinia Virus Gene *A46R*, Encoding for an Inhibitor of TLR Signalling, Is an Effective Approach to Enhance the Immunogenicity in Mice of the HIV/AIDS Vaccine Candidate NYVAC-C

**DOI:** 10.1371/journal.pone.0074831

**Published:** 2013-09-17

**Authors:** Beatriz Perdiguero, Carmen Elena Gómez, Mauro Di Pilato, Carlos Oscar S. Sorzano, Julie Delaloye, Thierry Roger, Thierry Calandra, Giuseppe Pantaleo, Mariano Esteban

**Affiliations:** 1 Department of Molecular and Cellular Biology, Centro Nacional de Biotecnología, Consejo Superior de Investigaciones Científicas (CSIC), Madrid, Spain; 2 Biocomputing Unit, Centro Nacional de Biotecnología, Consejo Superior de Investigaciones Científicas (CSIC), Madrid, Spain; 3 Infectious Diseases Service, Department of Medicine, Centre Hospitalier Universitaire Vaudois and University of Lausanne, Lausanne, Switzerland; 4 Division of Immunology and Allergy, Department of Medicine, Centre Hospitalier Universitaire Vaudois and University of Lausanne, Lausanne, Switzerland; Imperial College London, United Kingdom

## Abstract

Viruses have developed strategies to counteract signalling through Toll-like receptors (TLRs) that are involved in the detection of viruses and induction of proinflammatory cytokines and IFNs. Vaccinia virus (VACV) encodes A46 protein which disrupts TLR signalling by interfering with TLR: adaptor interactions. Since the innate immune response to viruses is critical to induce protective immunity, we studied whether deletion of *A46R* gene in a NYVAC vector expressing HIV-1 Env, Gag, Pol and Nef antigens (NYVAC-C) improves immune responses against HIV-1 antigens. This question was examined in human macrophages and in mice infected with a single *A46R* deletion mutant of the vaccine candidate NYVAC-C (NYVAC-C-ΔA46R). The viral gene *A46R* is not required for virus replication in primary chicken embryo fibroblast (CEF) cells and its deletion in NYVAC-C markedly increases TNF, IL-6 and IL-8 secretion by human macrophages. Analysis of the immune responses elicited in BALB/c mice after DNA prime/NYVAC boost immunization shows that deletion of *A46R* improves the magnitude of the HIV-1-specific CD4 and CD8 T cell immune responses during adaptive and memory phases, maintains the functional profile observed with the parental NYVAC-C and enhances anti-gp120 humoral response during the memory phase. These findings establish the immunological role of VACV *A46R* on innate immune responses of macrophages *in vitro* and antigen-specific T and B cell immune responses *in vivo* and suggest that deletion of viral inhibitors of TLR signalling is a useful approach for the improvement of poxvirus-based vaccine candidates.

## Introduction

The search for a safe and effective HIV vaccine able to elicit long-lasting protective immunity has encouraged the development of recombinant live vaccine candidates with good safety and immunogenicity profiles. The Thai phase III clinical trial (RV144) using the recombinant poxvirus vector ALVAC and the protein gp120 in a prime-boost strategy and showing a 31.2% protection against HIV infection [1], has raised considerable interest in the use of improved attenuated poxvirus recombinants as HIV vaccine candidates. Among poxviruses, the highly attenuated vaccinia virus (VACV) strain NYVAC is under intense preclinical and clinical evaluation as a vaccine against emergent infectious diseases and cancer [2].

The NYVAC strain was derived from a plaque clone isolate of the Copenhagen vaccinia virus strain (VACV-COP) by the deletion of 18 open reading frames (ORFs) involved in virulence, pathogenesis and host range functions [3]. In spite of its limited replication in human and most mammalian cell types, NYVAC provides a high level of gene expression and induces antigen-specific immune responses when administered to animals and humans [2,4,5,6]. However, the vector still contains other immunomodulatory viral genes that may suppress host immunity, particularly genes encoding proteins that antagonize the innate immune response mediated by Toll-like receptor (TLR) signalling. The deletion of these immunomodulatory genes could be a strategy to further improve NYVAC-based vaccines with the aim to obtain enhanced magnitude, breadth, polyfunctionality and durability of the immune responses.

The sensing of viral pathogens and the subsequent innate immune responses triggered are critical to produce protective immunity. Cells of the innate immune system detect viruses through the recognition of specific pathogen-associated molecular patterns (PAMPs) by pattern recognition receptors (PRRs) [7,8,9,10], among which TLRs are the best characterized [11]. TLR3, TLR7/8 and TLR9 reside predominantly within the endosomes where they recognize viral nucleic acids being involved in the generation of potent antiviral responses [12] while viral glycoprotein products have been shown to interact with TLR2 and TLR4 expressed on the cell surface [13,14]. The implication of TLR2 in the induction of type I IFN in inflammatory monocytes following *in vivo* infection with VACV has been reported and depletion of these cells leads to elevated levels of VACV in ovaries of mice [15]. TLR2 signalling has also been shown to be important for clonal expansion and memory CD8 T cells formation following VACV infection [16] and in VACV-induced production of proinflammatory cytokines by murine denditic cells (DCs) [17]. The best known role of TLR4 is the detection of lipopolysaccharide (LPS) but this receptor is also involved in the immune response to viruses. For example, TLR4 has been reported to be protective in pulmonary VACV infection since mice deficient for TLR4 signalling showed enhanced viral replication, hypothermia and mortality compared to control animals [18]. Because TLRs are expressed both on specific nonimmune cells, such as epithelial cells at potential sites of entry, and on a variety of immune cells including macrophages, DCs, B cells and certain types of T cells, they play a key role in the defence against pathogens through the induction of proinflammatory cytokines and type I IFNs but also in shaping pathogen-specific humoral and cellular adaptive immune responses.

All TLRs are type I transmembrane glycoprotein receptors comprised of an extracellular N-terminal leucine-rich repeat (LRR) domain involved in ligand binding, a single transmembrane domain and an intracellular C-terminal domain, known as the Toll/IL-1 receptor (TIR) domain, which mediates the interaction and recruitment of various adaptor proteins to activate the downstream signalling pathway [19]. PAMP binding induces receptor homo- or heterodimerization [20,21] and this activated conformation of the receptor triggers the recruitment of TIR domain-containing adaptor proteins that connect downstream signalling molecules leading to the activation of transcription factors such as IFN regulatory factors (IRFs) and NF-κB and the induction of type I IFNs and proinflammatory cytokines, respectively. Ligand recognition by TLRs induces the recruitment of five different adaptor proteins: Myeloid differentiation factor 88 (MyD88), MyD88-adaptor-like (Mal), TIR domain-containing adaptor protein-inducing IFN-β (TRIF), TRIF-related adaptor molecule (TRAM) and sterile α- and armadillo-motif-containing protein (SARM) [22]. Two major pathways can be activated by TLRs: the MyD88-dependent pathway, used by all TLRs except TLR3 [23] and the TRIF-dependent pathway, used by TLR3 and TLR4. TLR4 is the only receptor being able to signal via both pathways due to the differential use of two adaptors, TRAM and Mal. TLR4 uses TRAM to recruit TRIF and induce a type I IFN response via the TRIF-dependent pathway while the use of the coadaptor Mal to recruit MyD88 via the MyD88-dependent pathway induce a proinflammatory response [24]. Crystal structures of the TIR domains of TLR2 [25], TLR10 [26], interleukin-1 receptor accessory protein-like (IL-1RAPL) [27] and Mal [28,29] and NMR structure of the TIR domain of MyD88 [30] have been determined. These studies identified a conserved protruding BB loop between the βB strand and the αB helix, which is essential for functional TLR signalling [31,32,33,34,35].

Viruses have developed strategies to target TLR-mediated signalling to manipulate and evade the host innate immune response [36]. VACV encodes some intracellular negative regulators of TLR signalling including A46 [37], A52 [38], N1 [39], B14 [40], K7 [41] and C6 [42]. A46 was the first virally encoded protein identified to contain a TIR domain [37,43]. Through this domain, A46 binds directly to the TIR domain-containing adaptors MyD88, Mal, TRIF and TRAM, disrupting the formation of Receptor: Adaptor TIR interactions [37] and therefore inhibiting downstream signalling to MAPKs, NF-κB and IRF-3 and interfering with both proinflammatory and type I IFN responses [37]. However, A46 does not interact with SARM, which is a negative regulator of TLR signalling [37]. It has also been shown that A46 protein contributes to virulence since VACV *A46R* deletion mutant was attenuated in a murine intranasal model [37]. An 11 amino acid peptide derived from A46 (called VIPER) has been reported to specifically inhibit TLR4 responses by directly targeting Mal and TRAM [44] and that A46 binds to Mal via a Bcl-2-like α-helical dimer subdomain [45]. The molecular basis for A46 antagonism of TLR4 has been recently reported [46]. A46 has been shown to impair TLR4 signalling by targeting the conserved BB loop of TIR proteins and thereby disrupting Receptor: Adaptor TIR interactions [46].

Since VACV has been reported to be sensed by TLR2 [15,16,17], TLR4 [18], TLR2-TLR6-MyD88, MDA-5/IPS-1 and NALP3 inflammasome [47] and *A46R* targets the TIR domain of the adaptors MyD88, Mal, TRIF and TRAM [37], in the present study we have asked to what extent *A46R* impacts on the immune responses against VACV. This question was addressed with NYCAC-C, an attenuated poxvirus vector expressing HIV-1 Env and Gag-Pol-Nef (GPN) antigens from clade C [48], where *A46R* was deleted (NYVAC-C-ΔA46R). Specific innate, adaptive and memory immune responses to HIV-1 antigens were evaluated in human macrophages and in a BALB/c mouse model comparing the recombinant virus in the presence or absence of *A46R*. Our findings provided evidence for an immunomodulatory role of VACV A46 protein.

## Results

### Generation and *in vitro* characterization of NYVAC-C-ΔA46R deletion mutant

**Figure 1:**
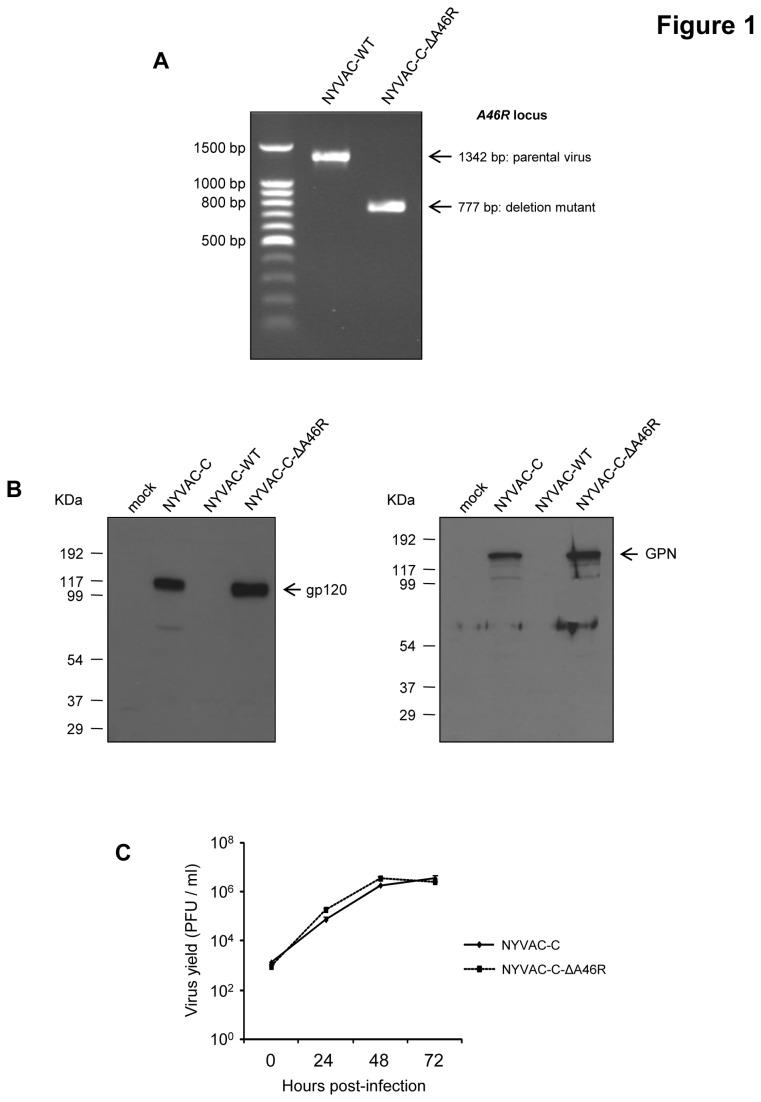
In vitro characterization of NYVAC-C-ΔA46R deletion mutant. (A) Confirmation of *A46R* gene deletion by PCR analysis. Viral DNA was extracted from BSC-40 cells infected with NYVAC-WT or NYVAC-C-ΔA46R at 5 PFU/cell. Primers LFA46R-Aat and RFA46R-Bam spanning *A46R* flanking sequences were used for PCR analysis of *A46R* locus. In parental NYVAC, a 1342 bp-product is obtained while in deletion mutant a unique 777 bp-product is observed. (B) Expression of HIV antigens by Western-blot. BSC-40 cells were mock-infected or infected at 5 PFU/cell with NYVAC-WT, NYVAC-C or NYVAC-C-ΔA46R. At 24 hours post-infection, cells were lysed in Laemmli buffer, cells extracts were fractionated by 8% SDS-PAGE and analyzed by Western-blot using a polyclonal anti-gp120 antibody or a polyclonal anti-gag p24 serum to evaluate the expression of gp120 and GPN proteins, respectively. (C) Analysis of virus growth of NYVAC-C-ΔA46R in CEF cells. Monolayers of CEF cells were infected with NYVAC-C or NYVAC-C-ΔA46R at 0.01 PFU/cell. At different times post-infection (0, 24, 48 and 72 hours), cells were collected and infectious viruses were quantified by immunostaining plaque assay in BSC-40 cells.

NYVAC-C-ΔA46R deletion mutant was generated as detailed under Materials and Methods using as parental virus the recombinant NYVAC-C that expresses the HIV-1 Env, Gag, Pol and Nef antigens from clade C [48] and following a strategy that allows the deletion of the gene of interest with no fluorescent marker included in the final deletion mutant. The correct deletion of *A46R* gene was confirmed by PCR using primers annealing in *A46R* flanking sequences. As shown in Figure 1A, *A46R* ORF was successfully deleted and no wild-type contamination was present in NYVAC-C-ΔA46R preparation. Analysis by Western-blot confirmed that the *A46R* deletion mutant expresses the HIV-1 proteins gp120 and GPN at the same level as the parental virus NYVAC-C (Figure 1B). Moreover, analysis by immunostaining showed that all virus plaques have immunoreactivity to anti-WR, anti-gp120 and anti-gag p24 antibodies (data not shown), demonstrating the stability of the antigens expressed by the *A46R* deletion mutant. To determine if deletion of *A46R* gene affects virus replication, we compared the growth kinetic of NYVAC-C-ΔA46R deletion mutant with its parental virus NYVAC-C in CEF cells. Figure 1C shows that the growth kinetics were similar between parental and deletion mutant, indicating that *A46R* gene is not required for virus replication in cultured cells and its deletion does not affect virus growth kinetics.

### NYVAC-C-ΔA46R up-regulates TNF, IL-6 and IL-8 production by human macrophages

**Figure 2:**
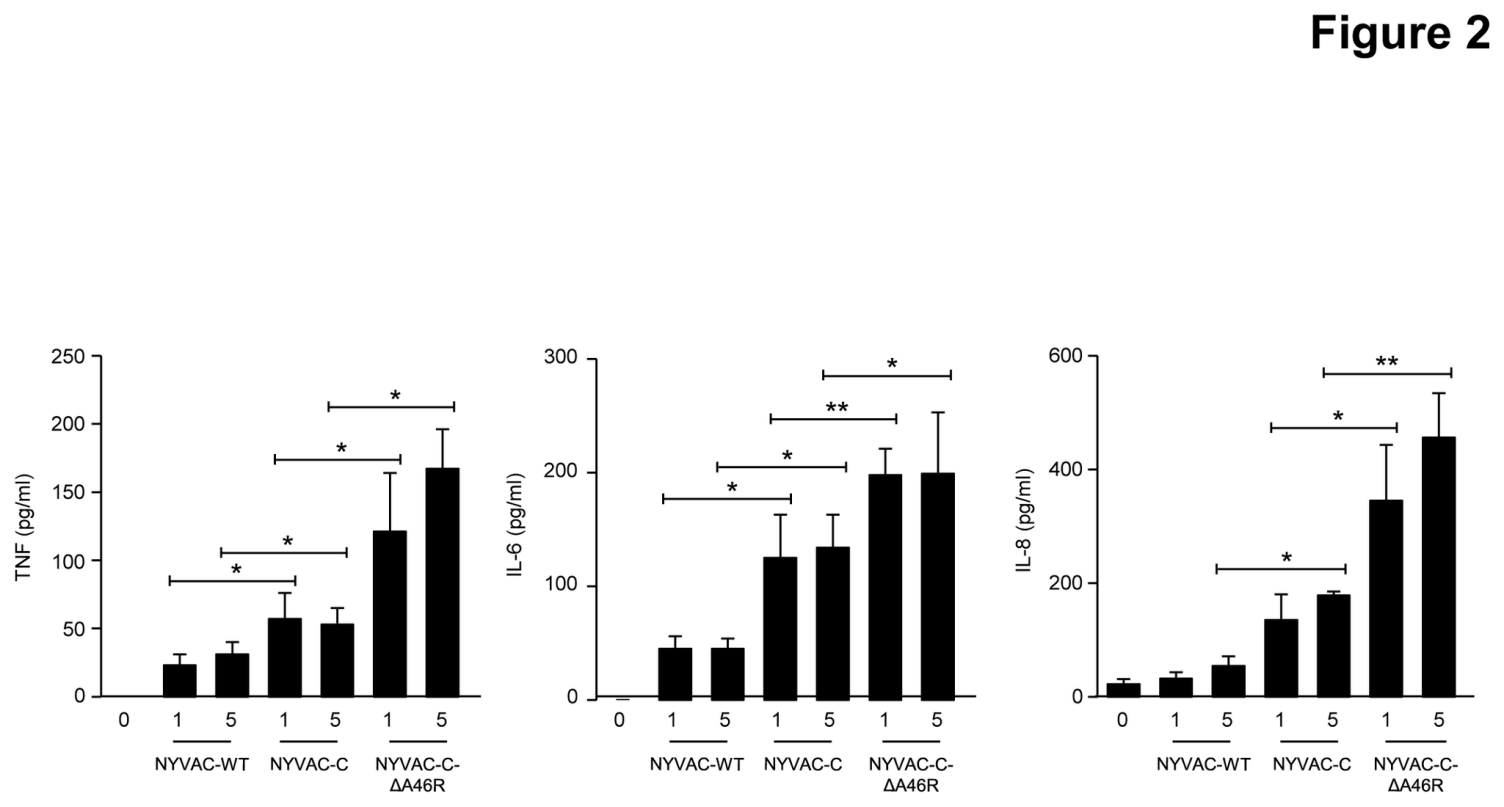
Deletion of *A46R* gene from NYVAC-C enhances innate immune responses. Human macrophages were mock-infected (0) or infected with NYVAC-WT, NYVAC-C or NYVAC-C-Δ46R (1 or 5 PFU/cell). 24 hours later, cell-free supernatants were collected to quantify the concentrations of TNF and IL-6 by bioassay and of IL-8 by ELISA. Data are means ± SD of duplicates and are representative of three independent experiments. * *p*<0.05, ** p<0.005.

To define whether *A46R* impairs the response of innate immune cells to NYVAC-C, we measured by ELISA the concentrations of proinflammatory cytokines and chemokines released by human THP-1 macrophages infected for 6 hours with 1 or 5 PFU/cell of NYVAC-WT, NYVAC-C or NYVAC-C-ΔA46R. Compared to NYVAC-WT and to NYVAC-C, the *A46R* deletion markedly up-regulated the production of TNF, IL-6 and IL-8 by THP-1 cells (Figure 2). Thus, the single deletion of *A46R* in the NYVAC-C genome triggers a stronger innate immune sensing than NYVAC-C, providing evidence for immune suppression by *A46R*.

Deletion of the viral gene *A46R* in NYVAC-C induces high, broad and polyfunctional HIV-1-specific T cell adaptive immune responses in BALB/c mice in heterologous prime/boost combination

To assay *in vivo* the effect of *A46R* gene deletion on the cellular immunogenicity against HIV-1 antigens, we analyzed the HIV-1-specific T cell adaptive immune responses elicited in mice by using a DNA prime/Poxvirus boost approach since it has been extensively reported that this heterologous immunization protocol is more immunogenic than either component alone to activate T cell responses to HIV-1 antigens [48,49,50].

BALB/c mice, 4 in each group, were immunized as described in Materials and Methods and adaptive T cell immune responses were measured 10 days after the last immunization by polychromatic intracellular cytokine staining (ICS) assay. Splenocytes from immunized animals were stimulated *ex vivo* for 6 hours with a panel of 464 peptides (15 mers overlapping by 11 amino acids) grouped in three pools: Env (112 peptides), Gag (121 peptides) and GPN (231 peptides) and stained with specific antibodies to identify T cell lineage (CD3, CD4 and CD8), degranulation (CD107a) and responding cells (IL-2, IFN-γ and TNF-α). The percentages of T cells producing IFN-γ and/or IL-2 and/or TNF-α established the overall CD4^+^ T cell responses whereas the percentages of T cells producing CD107a and/or IFN-γ and/or IL-2 and/or TNF-α determined the overall CD8^+^ T cell responses.

**Figure 3:**
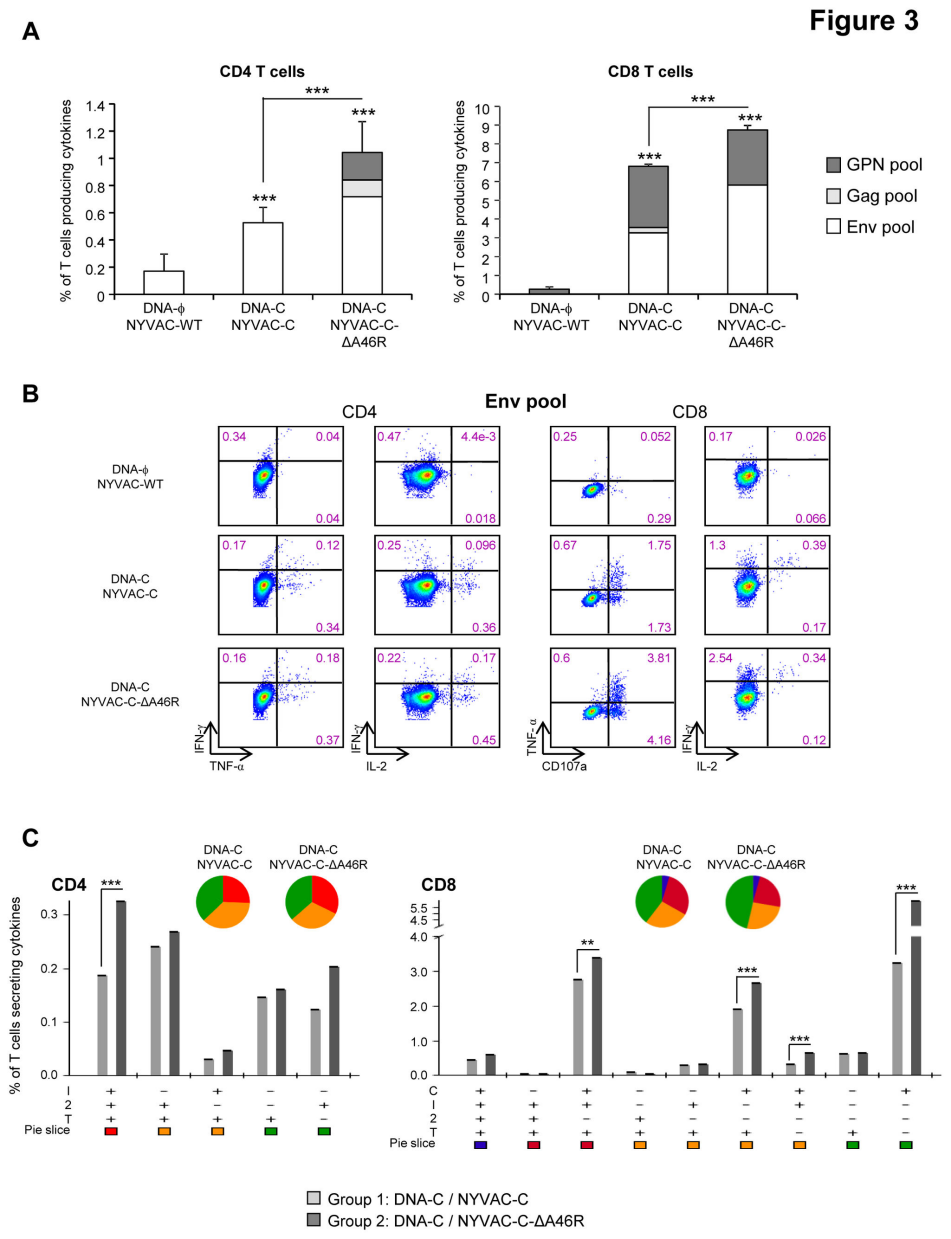
Adaptive HIV-specific T cell immune responses elicited by *A46R* deletion mutant in the spleen of BALB/c mice in heterologous prime/boost immunization protocol. (A) Magnitude of the vaccine-specific CD4 or CD8 T cell response. The HIV-specific CD4 or CD8 T cells were measured 10 days after the last immunization by ICS assay following stimulation of splenocytes derived from immunized animals (n=4) with the different HIV peptide pools. The total value in each group represents the sum of the percentages of CD4^+^ or CD8^+^ T cells secreting IFN-γ and/or IL-2 and/or TNF-α (CD4) or CD107a and/or IFN-γ and/or IL-2 and/or TNF-α (CD8) against all HIV peptide pools. All data are background-subtracted. *** *p*<0.001. *p* value indicates significantly higher responses compared to parental group or between DNA-C/NYVAC-C-ΔA46R and DNA-C/NYVAC-C immunization groups. (B) Flow cytometry profiles of vaccine-induced CD4 or CD8 T cell responses against Env pool. (C) Functional profile of the adaptive HIV-specific CD4 or CD8 T cell response in the different immunization groups. The possible combinations of the responses are shown on the *x* axis, whereas the percentages of the functionally distinct cell populations within the total CD4 or CD8 T cell population are shown on the *y* axis. Combinations that did not contribute significantly to the functional profile are not shown. Responses are grouped and colour-coded on the basis of the number of functions. The non-specific responses obtained in the control group DNA-ϕ/NYVAC-WT were subtracted in all populations. ** p<0.005, *** *p*<0.001. *p* values indicate significantly higher responses compared to DNA-C/NYVAC-C immunization group.

As shown in Figure 3A, in both immunization groups DNA-C/NYVAC-C and DNA-C/NYVAC-C-ΔA46R the magnitudes of the HIV-1-specific CD4 or CD8 T cell responses, determined as the sum of the individual responses obtained for Env, Gag and GPN peptide pools, were significantly higher than those obtained in the control group DNA-ϕ/NYVAC-WT (p<0.001). Furthermore, the magnitudes of the HIV-1-specific CD4 or CD8 T cell responses in the group immunized with NYVAC-C-ΔA46R were significantly higher than those obtained in the group DNA-C/NYVAC-C (*p*<0.001). In animals immunized with the parental NYVAC-C, the CD4^+^ T cell response was only directed against the Env pool while in the group boosted with the NYVAC-C-ΔA46R deletion mutant this response was mainly mediated by Env pool but the response against Gag and GPN peptide pools also contributes to the total HIV-1-specific CD4 T cell response. On the other hand, the CD8^+^ T cell responses were higher in magnitude and *A46R* gene deletion induced a significant enhancement in the magnitude of the CD8^+^ T cell responses against the Env pool (p<0.001) whereas the anti-GPN response was maintained. Representative functional profiles of Env-specific CD4 or CD8 T cell responses are shown in Figure 3B.

The quality of a T cell response can be characterized in part by the pattern of cytokine production and by the cytotoxic potential. On the basis of the analysis of IFN-γ, IL-2 and TNF-α secretion, as well as the study of CD107a expression on the surface of activated T cells as an indirect marker of cytotoxicity, 8 HIV-specific CD4 T cell populations and 16 HIV-specific CD8 T cell populations were identified. Vaccine-induced CD4 T cell responses were highly polyfunctional in both DNA-C/NYVAC-C and DNA-C/NYVAC-C-ΔA46R groups, with more than 60% of CD4 T cells exhibiting two or three functions. CD4 T cells producing IFN-γ+IL-2+TNF-α, IL-2+TNF-α or only TNF-α or IL-2 were the most representative populations induced by the parental NYVAC-C and the *A46R* deletion mutant, although the percentages of cells producing cytokines were low (Figure 3C). The HIV-1-specific CD8 T cell responses, higher in magnitude, were also polyfunctional in both immunization groups, with more than 50% of CD8^+^ T cells exhibiting two, three or four functions. CD8^+^ T cells producing CD107a+ IFN-γ+TNF-α, CD107a+ TNF-α or only CD107a were the most representative populations induced by the parental NYVAC-C and NYVAC-C-ΔA46R deletion mutant (Figure 3C).

Overall, these results indicate that deletion of *A46R* gene from NYVAC-C genome improved the magnitude of the HIV-1-specific adaptive CD4 and CD8 T cell immune responses and maintained the polyfunctional profile observed with the parental NYVAC-C. Since the contribution of DNA priming is the same for NYVAC-C and NYVAC-C-ΔA46R immunization groups, the differences observed should be attributed to the *A46R* deletion.

### Deletion of the viral gene *A46R* impacts on the HIV-1-specific CD8 T cell memory phase of the immune response

Phenotypic analysis of memory vaccine-induced T cell immune responses was performed by polychromatic ICS assay 53 days after the last immunization. Splenocytes from immunized mice were stimulated *ex vivo* for 6 hours with the HIV-1 peptide pools Env, Gag and GPN and stained with specific antibodies to identify T cell lineage (CD3, CD4 and CD8), degranulation (CD107a), responding cells (IL-2, IFN-γ and TNF-α) as well as memory stages (CD127 and CD62L).

**Figure 4:**
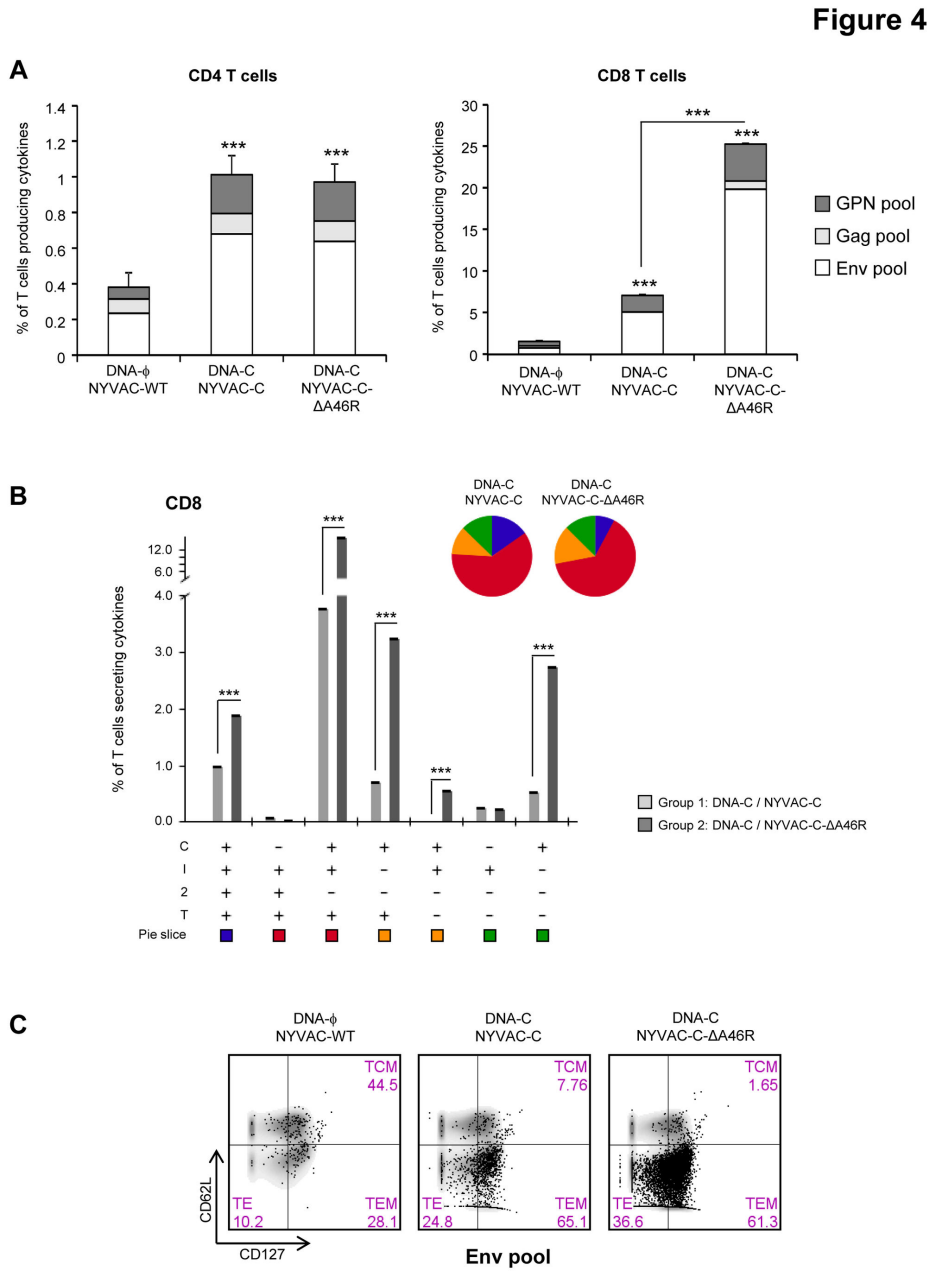
Memory HIV-specific T cell immune responses elicited by *A46R* deletion mutant in the spleen of BALB/c mice after prime/boost immunization. (A) Magnitude of the vaccine-specific CD4 or CD8 T cell responses. The HIV-specific CD4 or CD8 T cells were measured 53 days after the last immunization by ICS assay following stimulation of splenocytes derived from immunized animals (n=4) with the different HIV peptide pools. The total value in each group represents the sum of the percentages of CD4^+^ or CD8^+^ T cells secreting IFN-γ and/or IL-2 and/or TNF-α (CD4) or CD107a and/or IFN-γ and/or IL-2 and/or TNF-α (CD8) against all HIV peptide pools. All data are background-subtracted. *** *p*<0.001. *p* value indicates significantly higher responses compared to parental group or between DNA-C/NYVAC-C and DNA-C/NYVAC-C-ΔA46R immunization groups. (B) Functional profile of the memory HIV-specific CD8 T cell response in the different immunization groups. The possible combinations of the responses are shown on the *x* axis, whereas the percentages of the functionally distinct cell populations within the total CD8 T cell population are shown on the *y* axis. Combinations that did not contribute significantly to the functional profile are not shown. Responses are grouped and colour-coded on the basis of the number of functions. *** *p*<0.001. *p* values indicate significantly higher responses compared to DNA-C/NYVAC-C immunization group. (C) Phenotypic profile of memory HIV-specific CD8 T cells. Representative FACS plots showing the percentage of Env-specific CD8 T cells with central memory (TCM; CD127^+^CD62L^+^), effector memory (TEM; CD127^+^CD62L^-^) or effector (TE; CD127^-^CD62L^-^) phenotype.

The magnitudes of the memory HIV-1-specific CD4 or CD8 T cell responses, determined as the sum of the individual responses obtained for Env, Gag and GPN peptide pools, were significantly higher in the groups boosted with the parental NYVAC-C or with the NYVAC-C-ΔA46R deletion mutant than in the control group immunized with NYVAC-WT (p<0.001) (Figure 4A).

The magnitude of the HIV-1-specific CD4 T cell response in the group immunized with DNA-C/NYVAC-C-ΔA46R was similar to that obtained in the group DNA-C/NYVAC-C and in both cases it was mainly directed against Env. On the other hand, the CD8^+^ T cell responses were higher in magnitude and *A46R* gene deletion clearly induced a significant enhancement in the magnitude of the CD8^+^ T cell responses against Env and GPN (p<0.001). Representative functional profiles of Env-induced CD8 T cell responses are shown in Figure S1.

HIV-specific CD8 T cell responses were polyfunctional in both immunization groups with 75% of CD8 T cells exhibiting two, three or four functions. CD8 T cells producing CD107a+ IFN-γ+TNF-α, CD107a+ IFN-γ+IL-2+TNF-α, CD107a+ TNF-α or only CD107a were the most representative populations induced (Figure 4B).

Since previous studies have shown that CD127 and CD62L define functionally distinct populations of memory antigen-specific T cells [51], we characterized the differentiation stages of the responding CD8 T cells into central memory (TCM; CD127^+^CD62L^+^), effector memory (TEM; CD127^+^CD62L^-^) or effector (TE; CD127^-^CD62L^-^) populations. As shown in Figure 4C, about 60% of the HIV-specific CD8 T cells were of TEM phenotype in the DNA-C/NYVAC-C and DNA-C/NYVAC-C-ΔA46R groups.

Overall, these results indicate that deletion of *A46R* gene from NYVAC-C genome improved the magnitude of the HIV-1-specific memory CD8 T cell immune response and maintained the polyfunctional profile and memory differentiation pattern observed with the parental NYVAC-C.

### Deletion of the viral gene *A46R* in NYVAC-C enhances the anti-gp120 humoral response during the memory phase

**Figure 5:**
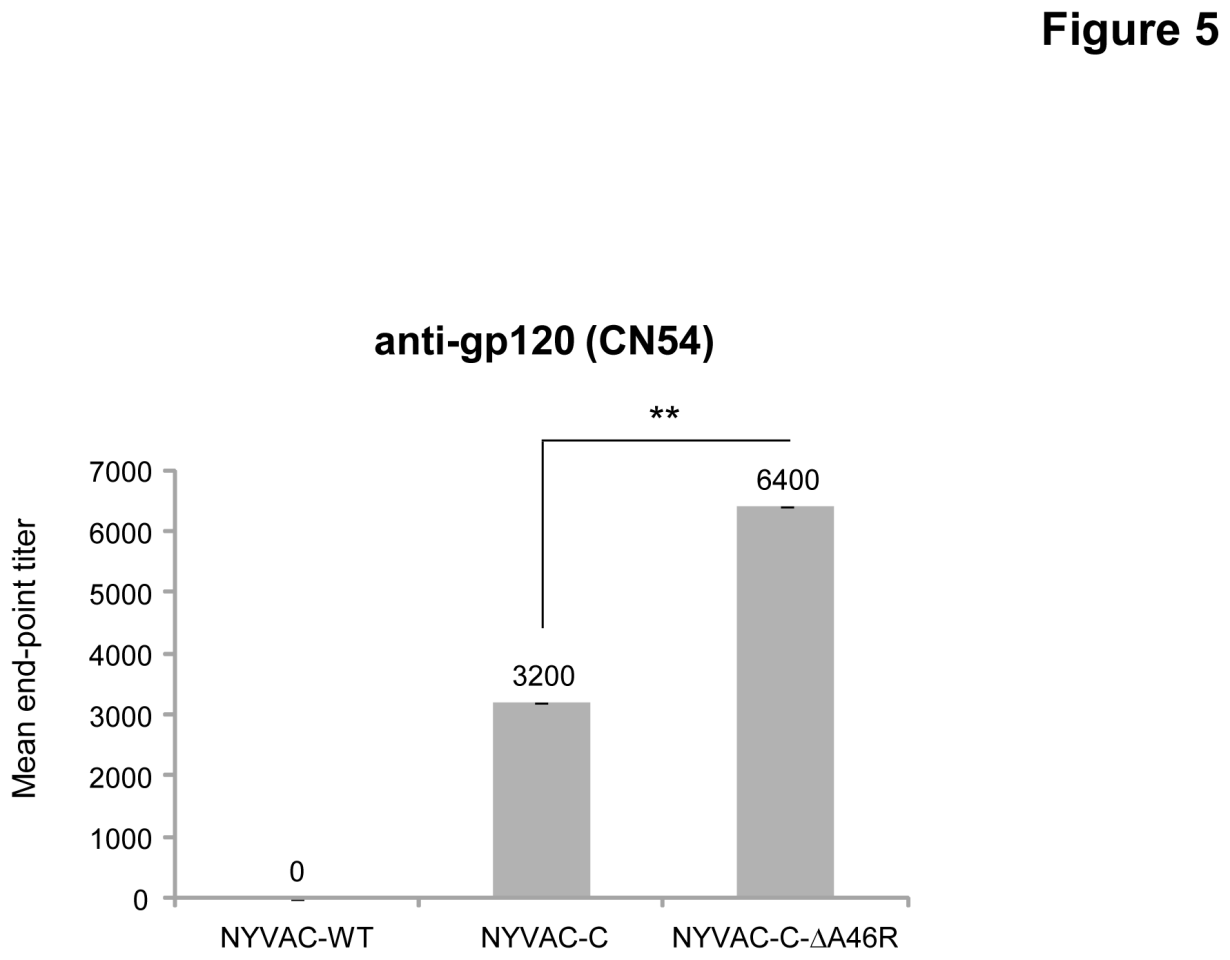
Memory humoral immune response elicited by the *A46R* deletion mutant against HIV-1 gp120 protein. Levels of Env-specific IgG binding antibodies were measured in serum from naïve and immunized mice at day 68. The values represent the mean antibodies titer for each group. ** p<0.005.

Since cells infected with NYVAC-C release monomeric gp120 [48], we also evaluated the impact of the deletion of viral gene *A46R* on the humoral response at day 68. We quantified by ELISA the Env-specific IgG titers against the purified gp120 protein from the HIV-1 isolate CN54 (clade C). As shown in Figure 5, the IgG titer obtained in the pool of sera of animals immunized with NYVAC-C-ΔA46R is significantly higher (p<0.005) than the titer obtained in the sera of animals immunized with NYVAC-C indicating that deletion of the viral gene *A46R* enhances the humoral response induced in mice during the memory phase.

### Deletion of the viral gene *A46R* impacts on the anti-vector CD8 T cell adaptive and memory phases of the immune response

**Figure 6:**
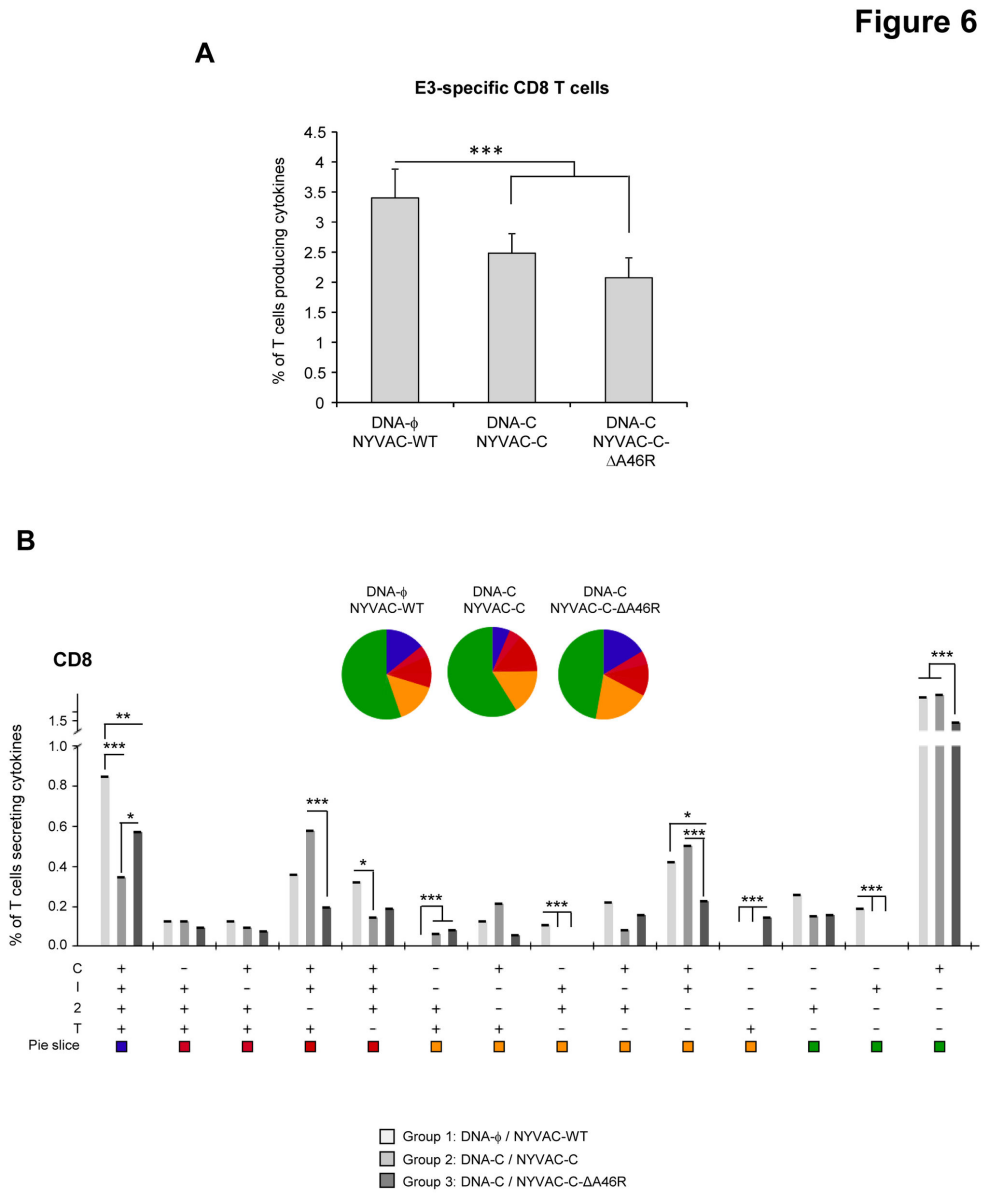
Adaptive VACV vector-specific T cell immune responses elicited by *A46R* deletion mutant in the spleen of BALB/c mice after prime/boost immunization. (A) Magnitude of the VACV-specific CD8 T cell response. The VACV-specific CD8 T cells were measured 10 days after the last immunization by ICS assay following stimulation of splenocytes derived from immunized animals (n=4) with VACV E3 peptide. The total value in each group represents the sum of the percentages of CD8^+^ T cells secreting CD107a and/or IFN-γ and/or IL-2 and/or TNF-α against E3 peptide. All data are background-subtracted. *** *p*<0.001. *p* value indicates significantly higher response compared to DNA-C/NYVAC-C and DNA-C/NYVAC-C-ΔA46R immunization groups. (B) Functional profile of the VACV-specific CD8 T cell response in the different immunization groups. The possible combinations of the responses are shown on the *x* axis, whereas the percentages of the functionally distinct cell populations within the total CD8 T cell population are shown on the *y* axis. Combinations that did not contribute significantly to the functional profile are not shown. Responses are grouped and colour-coded on the basis of the number of functions. * p<0.05, ** p<0.005, *** *p*<0.001.

**Figure 7:**
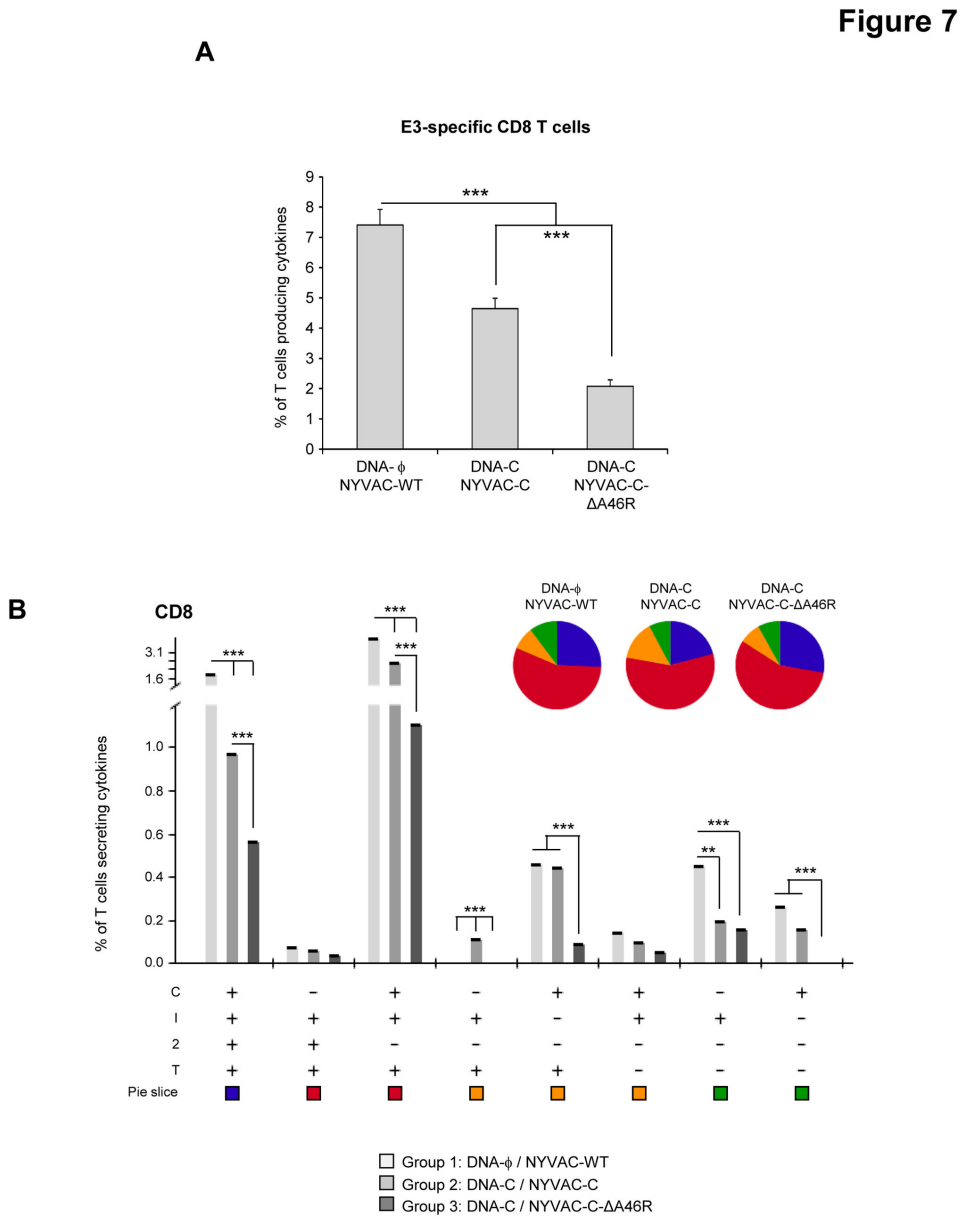
Memory VACV vector-specific T cell immune responses elicited by *A46R* deletion mutant in the spleen of BALB/c mice after prime/boost immunization. (A) Magnitude of the VACV-specific CD8 T cell response. The VACV-specific CD8 T cells were measured 53 days after the last immunization by ICS assay following stimulation of splenocytes derived from immunized animals (n=4) with VACV E3 peptide. The total value in each group represents the sum of the percentages of CD8^+^ T cells secreting CD107a and/or IFN-γ and/or IL-2 and/or TNF-α against E3 peptide. All data are background-subtracted. *** *p*<0.001. *p* values indicate significantly higher response compared to DNA-C/NYVAC-C and DNA-C/NYVAC-C-ΔA46R immunization groups or between DNA-C/NYVAC-C and DNA-C/NYVAC-C-ΔA46R groups. (B) Functional profile of the VACV-specific CD8 T cell response in the different immunization groups. The possible combinations of the responses are shown on the *x* axis, whereas the percentages of the functionally distinct cell populations within the total CD8 T cell population are shown on the *y* axis. Combinations that did not contribute significantly to the functional profile are not shown. Responses are grouped and colour-coded on the basis of the number of functions. ** p<0.005, *** *p*<0.001.

Vaccine-induced anti-vector T cell immune response was measured 10 and 53 days after the last immunization by polychromatic ICS assay. Splenocytes from immunized animals were stimulated *ex vivo* for 6 hours with VACV E3 peptide, which is specific for CD8 T cells [52]. During the adaptive phase of the immune response, the magnitude of the E3-specific CD8 T cell response was significantly lower in the DNA-C/NYVAC-C and DNA-C/NYVAC-C-ΔA46R immunized groups than in the control group immunized with DNA-ϕ/NYVAC-WT (p<0.001) (Figure 6A). No statistical differences were observed between the DNA-C/NYVAC-C and DNA-C/NYVAC-C-ΔA46R groups. E3-specific CD8 T cell responses were polyfunctional in all the immunization groups with almost 50% of CD8^+^ T cells exhibiting two, three or four functions. CD8 T cells producing only CD107a were the most representative population induced (Figure 6B). During the memory phase, the magnitude of the E3-specific CD8 T cell response in both immunization groups DNA-C/NYVAC-C and DNA-C/NYVAC-C-ΔA46R was significantly lower than that obtained in the control group DNA-ϕ/NYVAC-WT (p<0.001) and the magnitude of the E3-specific CD8 T cell response observed in the group DNA-C/NYVAC-C-ΔA46R was significantly lower than that obtained in the group DNA-C/NYVAC-C (p<0.001) (Figure 7A). E3-specific CD8 T cell responses were polyfunctional in all the immunization groups with almost 90% of CD8^+^ T cells exhibiting two, three or four functions. CD8 T cells producing CD107a+ IFN-γ+TNF-α or CD107a+ IFN-γ+IL-2+TNF-α were the most representative populations induced (Figure 7B). Overall, these results indicate that deletion of *A46R* gene from NYVAC-C genome reduced the magnitude of the VACV E3-specific adaptive and memory CD8 T cell immune response but maintained the polyfunctional profile observed with the parental NYVAC-C. Since adaptive and memory immune responses to HIV antigens were enhanced by the *A46R* deletion mutant (Figures 3 and 4), the reduced T cell immune response induced by the E3 peptide indicates an immunodominance of HIV antigens.

## Discussion

Development of non-replicating VACV vectors with enhanced immunogenicity against foreign expressed antigens is a major goal in the poxvirus field, aiming at the application of these vectors as HIV/AIDS vaccines. This is in view of the restricted immunogenicity triggered in clinical trials by the parental vectors expressing HIV antigens, like MVA, NYVAC, canarypox and fowlpox [53]. In fact, the reduced efficacy against HIV infection, 31.2%, of the non-replicating canary poxvirus vector combined with gp120 protein in the RV144 clinical trial [1], highlighted the need of novel poxvirus vectors with improved immunogenicity. With regard to non-replicating poxvirus vectors, different strategies have been pursued to enhance their potency, like the combination of heterologous vectors, use of co-stimulatory molecules and disruption of viral genes encoding immunosuppressive molecules [53]. The latter strategy provides the additional advantage that the immunomodulatory role of a viral gene can be easily quantified in an organism.

A number of MVA deletion mutants in viral immune modulators have been generated to date and tested in mice [54,55,56,57,58] and macaques [59,60]. These studies have shown that MVA recombinant viruses with a single deletion of viral genes encoding inhibitors of type 1 IFN signalling pathway (*C6L* [55]), apoptosis (*F1L* [56]), IL-18 binding protein (*C12L* [57]) or the uracyl-DNA glycosylase gene (*UDG* [60]), enhanced the overall immune responses to HIV-1 antigens. The HIV-1-specific CD4 and CD8 T cell immune responses were further increased by MVA vectors with deletions of two (*A41L*/*B16R* [54]; or *C6L*/*K7R*; Garcia-Arriaza, submitted) or four [IL-18 binding protein (MVA008L; *C12L*), Toll/IL-1 receptor homolog (MVA159R; *A46R*), CC-chemokine binding protein (MVA153L; *B7R*) and secreted IL-1β receptor (MVA184R; *B16R*)] immunomodulatory genes [59], while an additional fifth deletion of the uracyl-DNA glycosylase gene (MVA101R) decreased the responses [59]. Similarly, NYVAC vectors with single or double deletions in VACV genes *B19R* and *B8R* encoding type I and type II IFN binding proteins, respectively, increased the immune responses to HIV antigens in the mouse model [61].

In an effort to uncover the role of VACV genes as immune modulators and search for potential applications of these vectors in the development of optimized vaccines, in this investigation we showed that deletion of the viral TLR inhibitor *A46R* gene in the NYVAC-C genome has no effect on the replication capacity of the virus in CEF cells but triggers expression of immunoregulatory genes in infected macrophages. NYVAC-C also enhances, though to a lesser extent, the production of these proinflammatory cytokines and chemokines compared to NYVAC-WT indicating that the expression of HIV-1 antigens has an effect on innate immune cells. The impact on antigen-presenting cells of the expression of HIV antigens from an attenuated poxvirus vector has been previously reported by using microarray technology in human dendritic cells infected with an MVA-based recombinant virus expressing gp120 and GPN from clade B [62].

Significantly, in mice immunized following a DNA prime/NYVAC boost protocol, the deletion mutant NYVAC-C-ΔA46R enhanced HIV-specific T cell immune responses. Both CD4 and CD8 T cells specific for HIV antigens were activated. In the adaptive phase, the magnitudes of the HIV-1-specific CD4 or CD8 T cell responses in the group immunized with NYVAC-C-ΔA46R were significantly higher than those obtained in the group DNA-C/NYVAC-C (*p*<0.001), maintaining the polyfunctional profile observed with the parental NYVAC-C. In the memory phase, deletion of *A46R* gene from NYVAC-C genome improved again the magnitude of the HIV-1-specific memory CD8 T cell immune response, while both the polyfunctional profile and memory differentiation pattern observed were similar as those obtained with the parental NYVAC-C. The main phenotype of the memory response was TEM, which is of immunological relevance as this phenotype has been correlated with protection in the macaque-SIV model [63,64].

This enhanced HIV-specific T cell immune response is in contrast with the lack or reduced effect of *A46R* deletion on VACV E3-specific T cell responses during adaptive or memory phases of the immune response, respectively (Figures 6 and 7). The absence or decrease of immune stimulatory effect observed when E3 was used to stimulate mouse splenocytes in comparison with the increased responses against HIV antigens is likely to be related to the immune dominance of the HIV antigens versus the viral E3 peptide and such immunodominance may be due to the effect of the priming with a DNA encoding the HIV-1 antigens and also to the fact that NYVAC-C expresses E3 under its natural early promoter while the HIV antigens are expressed at early and late times from a strong synthetic early/late promoter. Since both explanations can be applied to NYVAC-C or NYVAC-C-ΔA46R-induced immune responses, the lower E3-specific CD8 T cell adaptive and memory immune responses elicited by NYVAC-C-ΔA46R deletion mutant compared with that induced by NYVAC-C are inversely correlated with the higher HIV-1-specific CD8 T cell responses triggered by the *A46R* deletion mutant. A similar trend for E3 response in relation to foreign expressed antigens has been observed for other recombinant VACV vectors [65]. Therefore, a reduction of immune responses to NYVAC-C-ΔA46R vector antigens has the additional vaccine advantage that HIV antigens are favoured over viral antigens, thus enhancing the specific immune responses to HIV.

Since humoral response against HIV antigens has been described to be important for protection against HIV acquisition [1], we also evaluated the presence of anti-gp120 antibodies in the serum of immunized animals. This analysis showed an enhanced anti-gp120 humoral response in the mice immunized with NYVAC-C-ΔA46R deletion mutant suggesting that the deletion of *A46R* gene is also able to modulate positively the humoral response against gp120.

How deletion of *A46R* impacts on the immune response of NYVAC-C? As previously described, A46 impairs TLR signalling by targeting the TIR domain of the adaptors MyD88, Mal, TRIF and TRAM disrupting Receptor: Adaptor TIR interactions [37]. Hence, deleting *A46R* in NYVAC restores TLR signalling upon viral infection, enhancing the expression of proinflammatory molecules, which in turn will enhance T cell activation. According to the intraperitoneal route used in the present study, the effect of *A46R* gene deletion on immunogenicity against HIV-1 antigens should be explained by the effect of TLR signalling restoration in the cell types present in the peritoneal cavity (mainly B cells, macrophages and granulocytes and, to a lesser extent, T cells [66]). In this context, the increased secretion of proinflammatory cytokines and chemokines by NYVAC-C-ΔA46R-infected macrophages could induce an enhanced recruitment of immature DCs and lymphocytes, generating an appropriate environment for the uptake and presentation of HIV-1 antigens to T cells. Immature NYVAC-C-ΔA46R-infected DCs can also migrate to the lymph nodes, maturing in route, and activate HIV-1-specific T cells enhancing the overall immunogenicity against HIV antigens. According to this, it has been previously reported that the total number of cells in the lungs of mice immunized intranasally with a VACV *A46R* deletion mutant was increased on day 2 post-infection compared with parental virus whereas on days 5 and 8 was reduced [37]. Since the main innate sensors of VACV vectors are TLR2 [15,16,17], TLR2-TLR6-MyD88, MDA-5/IPS-1 and NALP3 inflammasome [47] and *A46R* targets the TIR domain of the adaptors MyD88, Mal, TRIF and TRAM [37], our findings of enhanced production of TNF, IL-6 and IL-8 in conjunction with an increase in the magnitude of CD4 and CD8 T cell immune responses to HIV antigens and an enhanced gp120-specific humoral response, reveal that *A46R* plays an important role as immune modulator. This observation, in combination with the biochemical data on the mode of action of A46, establishes the immunological role of VACV *A46R* on T and B cell responses.

## Materials and Methods

### Ethics statement

The animal studies were approved by the Ethical Committee of Animal Experimentation (CEEA-CNB) of Centro Nacional de Biotecnologia (CNB-CSIC, Madrid, Spain) in accordance with national and international guidelines and with the Royal Decree (RD 1201/2005) (Permit numbers: 152/07 and 080030).

### Cells and viruses

African green monkey kidney cells (BSC-40; American Type Culture Collection, Manassas, VA) and primary chicken embryo fibroblast cells (CEF; Intervet, s.a, Salamanca, Spain) were grown in Dulbecco’s modified Eagle’s medium (DMEM) supplemented with 100 IU/ml of penicillin, 100 µg/ml of streptomycin and 10% newborn calf serum (NCS) for BSC-40 cells or 10% fetal calf serum (FCS) for CEF cells. The human monocytic THP-1 cells (American Type Culture Collection, Manassas, VA) were cultured in RPMI 1640 medium containing 2 mM L-glutamine, 50 µM 2-mercaptoethanol, 100 IU/ml of penicillin, 100 µg/ml of streptomycin and 10% FCS. Cells were maintained in a humidified air 5% CO_2_ atmosphere at 37°C. The poxvirus strains used in this work included the genetically attenuated vaccinia virus-based vector NYVAC-WT and the recombinant NYVAC-C expressing gp120 as a cell-released product and Gag-Pol-Nef as an intracellular polyprotein from the clade C CN54 HIV-1 isolate [48], used as the parental vector for the generation of the *A46R* deletion mutant. Virus infections were performed with 2% NCS or FCS. All viruses were grown in primary CEF cells, similarly purified through two 36% (w/v) sucrose cushions and the virus titers were determined by immunostaining plaque assay in BSC-40 cells as previously described [67]. The titer determinations of the different viruses were performed at least three times.

### Construction of plasmid transfer vector pGem-RG-A46R wm

The plasmid transfer vector pGem-RG-A46R wm, used for the construction of the recombinant virus NYVAC-C-ΔA46R, with *A46R* ORF deleted, was obtained by the sequential cloning of *A46R* recombination flanking sequences into the plasmid pGem-Red-GFP wm, containing dsRed2 and rsGFP genes as fluorescent markers, and previously described [68]. NYVAC genome was used as the template to amplify the left flank of *A46R* gene (432 bp) with oligonucleotides LFA46R-Aat (5´-CACGATGACGTCAGAGGAGTTAT-3´) (AatII site underlined) and LFA46R-Xba (5´-CGTATGTCTAGATTATTTTGCTGAG-3´) (XbaI site underlined). This left flank was digested with AatII and XbaI and cloned into plasmid pGem-Red-GFP wm previously digested with the same restriction enzymes to generate pGem-RG-LFsA46R wm (4939 bp). The repeated left flank of *A46R* gene (432 bp) was amplified by PCR from NYVAC genome with oligonucleotides LFA46R’-Eco (5´-CACGATGAATTCAGAGGAGTTAT-3´) (EcoRI site underlined) and LFA46R’-Cla (5´-CGTATGATCGATT TATTTTGCTGAG-3´) (Cla I site underlined), digested with EcoRI and Cla I and inserted into the EcoRI / Cla I-digested pGem-RG-LFsA46R wm to generate pGem-RG-LFdA46R wm (5330 bp). The right flank of *A46R* gene (360 bp) was amplified by PCR from NYVAC genome with oligonucleotides RFA46R-Cla (5´-CTGAGAATCGATAGGATGAATTTG-3´) (Cla I site underlined) and RFA46R-Bam (5´-ATTTAAGGATCCAGAACGGCAAC-3´) (BamHI site underlined), digested with Cla I and BamHI and inserted into the Cla I / BamHI-digested pGem-RG-LFdA46R wm. The resulting plasmid pGem-RG-A46R wm (5660 bp; Figure S2) was confirmed by DNA sequence analysis and directs the deletion of *A46R* gene from NYVAC-C genome.

### Construction of NYVAC-C-ΔA46R deletion mutant

The deletion mutant NYVAC-C-ΔA46R was constructed using dsRed2 and rsGFP as fluorescent markers. 3 x 10^6^ BSC-40 cells were infected with 0.01 PFU/cell of NYVAC-C and transfected 1 hour later with 6 µg DNA of plasmid pGem-RG-A46R wm using Lipofectamine (Invitrogen) according to the manufacturer’s recommendations. Forty-eight hours post-infection, the cells were harvested, lysed by freeze-thaw cycling, sonicated and used for recombinant virus screening. Deletion mutant was selected from progeny virus by consecutive rounds of plaque purification in BSC-40 cells during which plaques were screened for Red2/GFP fluorescence. In the first three passages, viruses from selected plaques expressed both fluorescent proteins, while in the next two passages viral progeny from selected plaques expressed only one fluorescent marker (Red2). In the last two passages (seven passages in total), viruses from selected plaques do not express any marker due to the loss of the fluorescent marker by homologous recombination within the repeated flanking DNA sequences. The resulting NYVAC-C-ΔA46R virus was expanded in BSC-40 cells and the crude preparation obtained was used for the propagation of the virus in large cultures of primary chicken fibroblasts (CEF) followed by virus purification through two 36% (w/v) sucrose cushions and titrated by immunoplaque assay in BSC-40 cells.

### PCR analysis of NYVAC-C-ΔA46R deletion mutant

To test the identity and purity of the recombinant virus NYVAC-C-ΔA46R, viral DNA was extracted from BSC-40 cells infected at 5 PFU/cell with NYVAC-WT or NYVAC-C-ΔA46R. Cell membranes were disrupted using sodium dodecyl sulphate (SDS) followed by proteinase K treatment (0.2 mg/ml proteinase K in 50 mM Tris-HCl pH 8, 100 mM EDTA pH 8, 100 mM NaCl and 1% SDS for 1 hour at 55°C) and phenol extraction of viral DNA. Primers LFA46R-Aat and RFA46R-Bam spanning *A46R* flanking regions were used for PCR analysis of *A46R* locus. The amplification reactions were carried out with Platinum Taq DNA polymerase (Invitrogen) according to the manufacturer’s recommendations. The correct sequence of deleted *A46R* locus was confirmed by DNA sequence analysis.

### Expression of HIV-1 proteins gp120 and GPN

To test the correct expression of HIV-1 antigens by the *A46R* deletion mutant, monolayers of BSC-40 cells were mock-infected or infected at 5 PFU/cell with NYVAC-WT, NYVAC-C or NYVAC-C-ΔA46R. At 24 hours post-infection, cells were lysed in Laemmli buffer, cells extracts fractionated by 8% SDS-PAGE and analyzed by Western-blot using the polyclonal anti-gp120 antibody (Centro Nacional de Biotecnología; diluted 1:3000) or the polyclonal anti-gag p24 serum (ARP 432, NIBSC, Centralised Facility for AIDS reagent, UK; diluted 1:1000) to evaluate the expression of gp120 and GPN proteins, respectively. The anti-rabbit-HRPO (SIGMA; diluted 1:5000) was used as secondary antibody. The immunocomplexes were detected by enhanced chemiluminescence (ECL, GE Healthcare).

### Analysis of virus growth

To determine virus growth profiles, monolayers of CEF cells grown in 12-well plates were infected in duplicate at 0.01 PFU/cell with NYCAC-C or NYVAC-C-ΔA46R deletion mutant. Following virus adsorption for 60 min at 37°C, the inoculum was removed. The infected cells were washed once with DMEM without serum and incubated with fresh DMEM containing 2% FCS at 37°C in a 5% CO_2_ atmosphere. At different times post-infection (0, 24, 48 and 72 hours), cells were harvested by scraping (lysates at 5 x 10^5^ cells/ml), freeze-thawed three times and briefly sonicated. Virus titers in cell lysates were determined by immunostaining plaque assay in BSC-40 cells using rabbit polyclonal anti-vaccinia virus strain WR (Centro Nacional de Biotecnología; diluted 1:1000), followed by anti-rabbit-HRPO (SIGMA; diluted 1:1000).

### Measurement of cytokine production by macrophages

THP-1 cells were differentiated into macrophages by treatment with 0.5 mM phorbol 12-myristate 13-acetate (PMA, Sigma-Aldrich) for 24 h. The medium was changed and cells were either mock-infected or infected with 1 or 5 PFU/cell of NYVAC-WT, NYVAC-C or NYVAC-C-Δ46R. Cell-free supernatants were collected after 6 hours to quantify the concentrations of TNF, IL-6 and IL-8. The concentrations of human IL-8 (BD Biosciences) in cell-culture supernatants were measured by ELISA as previously described [47]. TNF and IL-6 concentrations were measured by bioassay as described elsewhere [69].

### DNA vectors

The two DNA constructs expressing the HIV-1 _CN54_gp120 (pcDNA- _CN54_gp120) and HIV-1 _CN54_Gag-Pol-Nef (GPN) polyprotein (pcDNA- _CN54_GPN) have been previously reported [48]. Plasmids were purified using Maxi-prep purification kits (Qiagen) and diluted for injection in endotoxin-free PBS.

### Peptides

The HIV-1 peptide pools Gag-1, Gag-2, Env-1, Env-2, GPN-1, GPN-2, GPN-3 and NEF were provided by the EuroVacc Foundation and were previously described [48]. They spanned the HIV-1 Env, Gag, Pol and Nef antigens from clade C included in the immunogens as consecutive 15-mers overlapping by 11 amino acids. For immunological analyses we grouped the pools as follows: Env pool (Env-1+Env-2), Gag pool (Gag-1+Gag-2) and GPN pool (GPN-1+GPN-2+GPN-3+NEF). The VACV E3_140-148_ peptide (VGPSNSPTF; CNB), previously described as immunodominant epitope in BALB/c mice [52], was used to detect the anti-vector cellular immune response.

### Mouse immunization schedule

BALB/c mice (6-8 weeks old) were purchased from Harlan. For the heterologous DNA prime/NYVAC boost immunization protocol performed to assay the immunogenicity of NYVAC-C-ΔA46R deletion mutant, groups of animals (n=8) received 100 µg of DNA-C (50 µg of pcDNA- _CN54_gp120 + 50 µg of pcDNA- _CN54_GPN) or 100 µg of DNA-ϕ (100 µg of pcDNA) by intramuscular route (i.m.). Two weeks later, animals were immunized with 1 x 10^7^ PFU of NYVAC-WT, NYVAC-C or NYVAC-C-ΔA46R by intraperitoneal route (i.p.). Mice immunized with sham DNA (DNA-ϕ) followed by NYVAC-WT boost were used as control group. At 10 and 53 days after the last immunization, 4 mice in each group were sacrificed and spleens processed for Intracellular Cytokine Staining (ICS) assay to measure the adaptive and memory cellular immune responses against HIV-1 antigens, respectively. Two independent experiments have been performed for the different groups.

### Intracellular Cytokine Staining assay (ICS)

The magnitude, polyfunctionality and phenotype of the HIV-specific T cell responses were analyzed by ICS. After an overnight rest, 4 x 10^6^ splenocytes (depleted of red blood cells) were seeded on 96-well plates and stimulated during 6 hours in complete RPMI 1640 media supplemented with 10% FCS containing 1 µl/ml GolgiPlug (BD Biosciences), anti-CD107a-Alexa 488 (BD Biosciences) and 5 µg/ml of the different HIV peptide pools. At the end of the stimulation period, cells were washed, stained for the surface markers, fixed and permeabilized (Cytofix/Cytoperm Kit; BD Biosciences) and stained intracellularly using the appropriate fluorochromes. Dead cells were excluded using the violet LIVE/DEAD stain kit (Invitrogen). For functional analyses the following fluorochrome-conjugated antibodies were used: CD3-PE-CF594, CD4-APC-Cy7, CD8-V500, IFN-γ-PE-Cy7, IL-2-APC and TNF-α-PE (all from BD Biosciences). In addition, for phenotypic analyses the following antibodies were used: CD62L-Alexa 700 (BD Biosciences) and CD127-PerCP-Cy5.5 (eBioscience). Cells were acquired using a GALLIOS flow cytometer (Beckman Coulter). Analyses of the data were performed using the FlowJo software version 8.5.3 (Tree Star, Ashland, OR). The number of lymphocyte-gated events ranged between 1 x 10^5^ and 1 x 10^6^. After gating, Boolean combinations of single functional gates were then created using FlowJo software to determine the frequency of each response based on all possible combinations of cytokine expression or all possible combinations of differentiation marker expression. For each population, background responses detected in the non-stimulated control samples were subtracted from those detected in stimulated samples for every specific functional combination and the percentages of cells producing cytokines obtained in the DNA-ϕ/NYVAC-WT control populations were also subtracted in all the groups in order to remove the non-specific responses detected as background. Only positive responses are represented.

### Antibody measurement by ELISA

Binding antibodies to Env protein in serum were determined by enzyme-linked immunosorbent assay (ELISA) as previously described [48]. Serum samples from naïve and immunized mice were serially 2-fold diluted in duplicate and reacted against 2 µg/ml of the recombinant CN54gp120 purified protein (ARP683, HIV-1 CN54gp120 clade C; EU Programme EVA from the Centre for AIDS Reagents). The antibody titer of Env-specific IgG was defined as the last dilution of serum that resulted in 3 times the mean optical density at 450 nm of the naïve control.

### Data analysis and statistics

For the statistical analysis of ICS data, we used a novel approach that corrects measurements for the medium response (RPMI) and allows the calculation of confidence intervals and *p* values of hypothesis tests [54,70]. Only antigen responses values significantly higher than the corresponding RPMI are represented and the background for the different cytokines in the unstimulated controls never exceeded 0.05%. Analysis and presentation of distributions was performed using SPICE version 5.1, downloaded from http://exon.niaid.nih.gov [71]. Comparison of distributions was performed using a Student’s T test and a partial permutation test as described [71]. All values used for analyzing proportionate representation of responses are background-subtracted. For the statistical analysis of ELISA data, a 1-way ANOVA with Tukey’s honestly significant difference criterion as post-hoc analysis was performed.

## Supporting Information

Figure S1 **Profile of memory HIV-specific T cell immune responses elicited by *A46R* deletion mutant in the spleen of BALB/c mice after prime/boost immunization.** Flow cytometry profiles of vaccine-induced CD8 T cell responses against Env pool in splenocytes from immunized animals.
(TIF)

Figure S2 **Scheme of construction of the plasmid transfer vector pGem-RG-A46R wm.** The plasmid transfer vector pGem-RG-A46R wm was obtained by the sequential cloning of *A46R* recombination flanking sequences into the plasmid pGem-Red-GFP wm, containing dsRed2 and rsGFP genes as fluorescent markers. NYVAC genome was used as the template to amplify the left flank of *A46R* gene by PCR. This left flank was digested with AatII and XbaI and cloned into plasmid pGem-Red-GFP wm previously digested with the same restriction enzymes to generate pGem-RG-LFsA46R wm (4939 bp). The repeated left flank of *A46R* gene was amplified by PCR from NYVAC genome, digested with EcoRI and Cla I and inserted into the EcoRI / Cla I-digested pGem-RG-LFsA46R wm to generate pGem-RG-LFdA46R wm (5330 bp). The right flank of *A46R* gene was amplified by PCR from NYVAC genome, digested with Cla I and BamHI and inserted into the Cla I / BamHI-digested pGem-RG-LFdA46R wm. The resulting plasmid pGem-RG-A46R wm (5660 bp) directs the deletion of *A46R* gene from NYVAC-C genome.
(TIF)
