# Supplementary figures and images for: Deletion of the Vaccinia Virus Gene A46R, Encoding for an Inhibitor of TLR Signalling, Is an Effective Approach to Enhance the Immunogenicity in Mice of the HIV/AIDS Vaccine Candidate NYVAC-C
Source: PLoS One. 2013 Sep 17;8(9):e74831. doi: 10.1371/journal.pone.0074831 (PMC3775734; doi:10.1371/journal.pone.0074831)

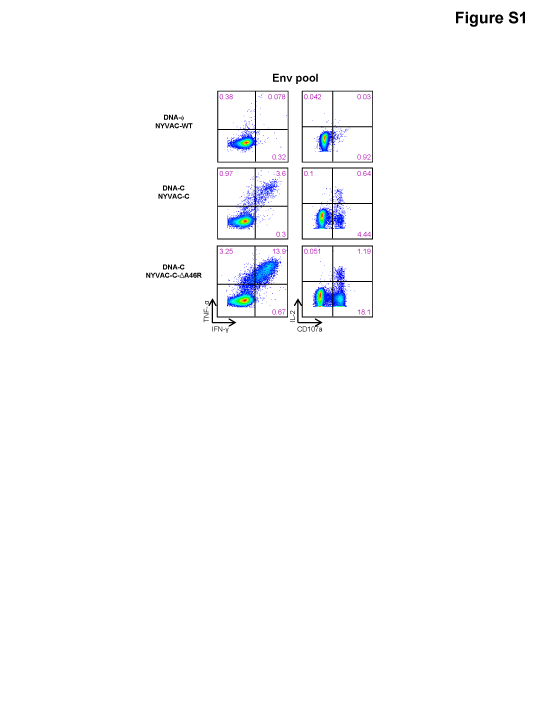

Supplement: Figure S1 — Profile of memory HIV-specific T cell immune responses elicited by A46R deletion mutant in the spleen of BALB/c mice after prime/boost immunization. Flow cytometry profiles of vaccine-induced CD8 T cell responses against Env pool in splenocytes from immunized animals. (TIF) [file pone.0074831.s001.tif]

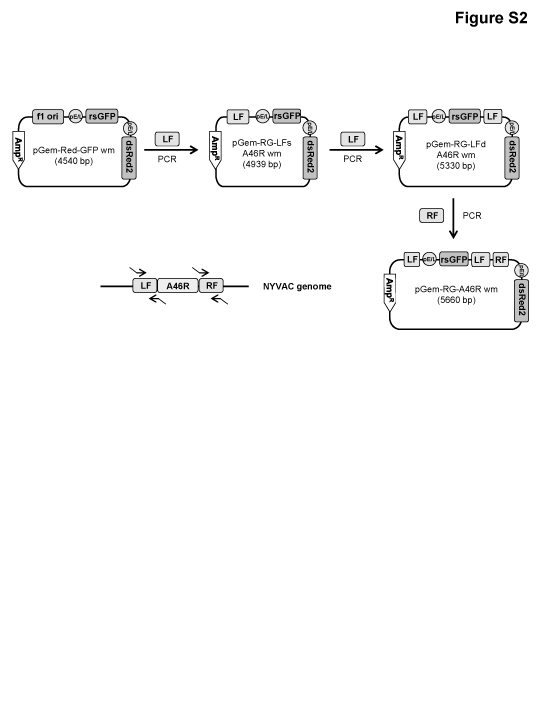

Supplement: Figure S2 — Scheme of construction of the plasmid transfer vector pGem-RG-A46R wm. The plasmid transfer vector pGem-RG-A46R wm was obtained by the sequential cloning of A46R recombination flanking sequences into the plasmid pGem-Red-GFP wm, containing dsRed2 and rsGFP genes as fluorescent markers. NYVAC genome was used as the template to amplify the left flank of A46R gene by PCR. This left flank was digested with AatII and XbaI and cloned into plasmid pGem-Red-GFP wm previously digested with the same restriction enzymes to generate pGem-RG-LFsA46R wm (4939 bp). The repeated left flank of A46R gene was amplified by PCR from NYVAC genome, digested with EcoRI and Cla I and inserted into the EcoRI / Cla I-digested pGem-RG-LFsA46R wm to generate pGem-RG-LFdA46R wm (5330 bp). The right flank of A46R gene was amplified by PCR from NYVAC genome, digested with Cla I and BamHI and inserted into the Cla I / BamHI-digested pGem-RG-LFdA46R wm. The resulting plasmid pGem-RG-A46R wm (5660 bp) directs the deletion of A46R gene from NYVAC-C genome. (TIF) [file pone.0074831.s002.tif]
